# Comment on Rochmah et al. Economic Burden of Stroke Disease: A Systematic Review. *Int. J. Environ. Res. Public Health* 2021, *18*, 7552

**DOI:** 10.3390/ijerph19074095

**Published:** 2022-03-30

**Authors:** Summaiya Zareen Shaikh, Anant Patil, Mohammad Usman Ali, Ajit Dabholkar, Gianluca Rossetto

**Affiliations:** 1 Department of Neuro-Physiotherapy, The SIA College of Physiotherapy, Thane 421203, India; 2 Department of Pharmacology, Dr. D.Y. Patil Medical College, Nerul, Navi Mumbai 400706, India; anant.patil@dypatil.edu; 3 Medical Rehabilitation, College of Medical Sciences, University of Maiduguri, P.M.B 1069, Maiduguri 600104, Nigeria; usmanali.muhammad@yahoo.com; 4 Department of Sports-Physiotherapy, School of Physiotherapy, D.Y. Patil University, Navi Mumbai 400706, India; ajit.dabholkar@dypatil.edu; 5 Department of Information Engineering, University of Brescia, 25123 Brescia, Italy; g.rossetto001@unibs.it

We have read with great interest the recently published article titled “Economic Burden of Stroke Disease: A Systematic Review” by Rochmah and colleagues [1]. The systematic review enrolled 13 out of 9270 articles from four databases (Scopus, Science Direct, Proquest, and Springerlink Journals). The authors aimed to systematically review the economic burden of stroke on the economy of the country as reported. The findings of the review revealed that stroke results in a substantial economic burden because of long-term care of the patient including during hospitalization and post-discharge management related to rehabilitation. The high financial burden in stroke management justifies the need for the promotion of stroke awareness campaigns and preventive efforts by policymakers to achieve the practice of healthy lifestyles among people. We want to express a few points of interest regarding their study eligibility criteria and also the employed methodology of the systematic review.

The authors followed the PRISMA methodology for systematic review but have not excluded any type of study designs as stated [2]. Surprisingly, there are no exclusion criteria, and sufficient reasons were not provided as to why there were not any. The authors included all types of publications, study designs including prospective and retrospective ones, database analyses, mathematical models, surveys, and cost of illness (COI) studies published in 2015–2020 in the English language examining the economic burden (direct and indirect costs) in the stroke population. The admixture of all types of study designs might have enabled the authors to extract similar characteristics, but narrowing it down to a distinct inference was obscure. We feel that the exclusion could have been based on specific economic evaluation indicators (especially the QALY and DALY), as mentioned by the authors in the introduction. We extracted three studies (Refs. [3,4,5]) from the pre-specified eligibility criteria and the same databases as those mentioned in the systematic review (Table 1 and Table 2). The three studies were observational studies from the given period of 2015 to 2020 and measured the economic burden using QALY/DALY indicators [3,4,5]. A well-defined exclusion criterion enables the researcher to eliminate biases arising due to the missing outcome data. The ‘*missingness mechanism*’ reflects on the relationship between the true value of the reported data and the included studies [6]. Concerning the methodology, the assessment of 2814 articles for full-text screening can be an exhaustive task. The use of suitable software for screening abstracts and removing duplicates instead of Microsoft excel, thereby reducing the numbers for scrutiny with a low risk of manual error, could have helped in the process. Additionally, justification for excluding titles and abstracts was lacking, most likely due to the absence of stringent eligibility criteria.

Furthermore, the ‘*7 question quality assessment tool*’ from Gheorge’s study, as quoted, is used to evaluate the “economic” & “epidemiological” aspect of particular studies and not for assessing the quality of the studies [7]. We reviewed Gheorge’s study, thereby noting that the above method definitely aided in extracting the economic & epidemiological variables for systematic evaluation but failed to evaluate the overall quality of the included study. We suggest the inculcation of a valid and robust qualitative assessment tool according to the types of study designs. To enhance the quality, the risk of bias assessment tool helps in identifying the critical domains and sources of bias such as selection bias, attrition bias, and reporting bias, amongst others [8].

The WHO’s Global Burden Disease Data Impact sources tool aims at reducing the economic disease mortality by 2% every year by assessing the socioeconomic index (SDI) [9]. This implies that a society is a grouping of a mixture of households with varied incomes. By neglecting any one sector of society, especially the ‘*lower-middle*’ & ‘*low-income*’ regions or countries, policy enactment takes a backseat and ends up being biased. Lastly, in the table of characteristics of the systematic review (Table 1), it is interesting to note that economic burden has been studied in ‘*high income*’ and ‘*upper-middle income*’ countries. The major pitfall of the review is that, in the included articles, not a single study got extracted on ‘*lower-middle-income countries*’ or ‘*low–income*’ countries. This probably could have been due to the absence of stringent eligibility criteria, and the possibility of a meta-analysis could have arisen.

Nevertheless, the strong points of this systematic review are that it highlights the magnitude of economic losses (direct medical & non-medical costs, indirect costs) stressing the country’s economy. We congratulate the authors for their scientific contribution to the International Journal of Environmental Research and Public Health. We sincerely hope that they consider our suggestions as useful to future research on a similar topic.

## Figures and Tables

**Table 1:** Studies published between January 2015 and March 2020 (Not included in the Systematic Review).

Study	Database	Journal	Study Design	Population	Country	Sample Size
Ock, et al. [3]	PubMed, Science Direct, Scopus	Value in Health	Cross-sectional	Stroke	Korea	229,229
Persson, et al. [4]	PubMed, Springer Link	Qual Life Res	Cross-sectional	Stroke	Sweden	Cohabitant dyads of 247 stroke survivors aged 70 at stroke onset and 245 dyads of controls were included
Sudharsanan, et al. [5]	PubMed, Scopus	BMJ Open	Cross-sectional	Stroke	India	94,154 individuals; stroke prevalence information was based on a door-to-door evaluation of all 45,053 individuals from 39 of the 86 villages in the surveillance site.

**Table 2:** Studies using QALY/DALY indicators used to investigate the economic burden of stroke.

Study	Indicators	Objective	Result
Ock, et al. [3]	QALY	Estimating Quality-Adjusted Life-Year loss due to Noncommunicable Diseases in Korean Adults through to the year 2040	The largest QALY loss due to mortality was stroke (306,733), whereas the largest QALY loss due to morbidity was arthritis (502,513).
Persson, et al. [4]	QALY	To investigate whether the dependency of midlife stroke survivors had any long-term impact on their spouses’ QALY-weights.	Spouses of dependent stroke survivors (*n* = 50) reported a significantly lower mean QALY-weight of 0.69 in comparison to spouses of independent stroke survivors (*n* = 197) and spouses of controls, (*n* = 245), who both reported a mean QALY-weight of 0.77. The results from the regression analysis showed that the higher age of the spouse and dependency of the stroke survivor had a negative association with the spouses’ QALY-weights.
Sudharsanan, et al. [5]	DALY	To directly estimate disability-adjusted life years (DALYs) lost due to stroke in rural Gadchiroli, India and measure the contribution of mortality and disability to total DALYs lost.	229 stroke deaths among the total population of 94,154 individuals and 175 stroke survivors among the subpopulation of 45,053 individuals. An estimated 2984 DALYs were lost due to stroke per 100,000 person-years, with a higher burden among men compared with women (3142 vs. 2821 DALYs). Over three-fourths (80%) of the total DALYs lost due to stroke were between the ages of 30 and 70 years. YLL accounted for 98.9% of total DALYs lost.
